# Using Malaise traps for collecting Lepidoptera (Insecta), with notes on the preparation of Macrolepidoptera from ethanol

**DOI:** 10.3897/BDJ.7.e32192

**Published:** 2019-02-20

**Authors:** Olga Schmidt, Stefan Schmidt, Christoph L. Häuser, Axel Hausmann, Lien Van Vu

**Affiliations:** 1 SNSB-Zoologische Staatssammlung München, Munich, Germany SNSB-Zoologische Staatssammlung München Munich Germany; 2 Museum für Naturkunde – Leibniz-Institut für Evolutions-und Biodiversitätsforschung, Berlin, Germany Museum für Naturkunde – Leibniz-Institut für Evolutions-und Biodiversitätsforschung Berlin Germany; 3 Vietnam National Museum of Nature, VAST, Hanoi, Vietnam Vietnam National Museum of Nature, VAST Hanoi Vietnam

**Keywords:** Butterflies, collecting methods, macrolepidoptera, Malaise trap, microlepidoptera, monitoring, moths, preparation technique, rapid biodiversity assessment, sampling, trapping

## Abstract

The present paper deals with the potential of employing Malaise traps for collecting butterflies and moths for morphological analysis and presents a protocol for preparing Macrolepidoptera from Malaise trap samples that were preserved in ethanol. About 80 specimens of Lepidoptera, including Nymphalidae, Geometridae, Hesperiidae, Erebidae, Noctuidae, Pyralidae and Tortricidae, were mounted, following the protocol. All specimens with robust wings and contrasting wing patterns were well suited for the study of external morphology, regardless of the family. The specimens used in this study were collected in highland forest areas of central Vietnam with a little known entomofauna, as part of the German-Vietnamese biodiversity project 'VIETBIO'. The study offers new methodological approaches in an attempt to make the most of the material that was obtained using Malaise traps.

## Introduction

Our study presents the results of a capacity building training course that was part of the ongoing German-Vietnamese 'VIETBIO' project, in June 2018. The project aims at establishing the foundation for collaborative biodiversity projects through capacity building and biodiversity research. Amongst other methods, Malaise traps (see Malaise 1937; Townes 1962; Townes 1972; Achterberg 2009; Sheikh et al. 2016) were employed in the Bach Ma National Park in central Vietnam. Located within the transition area of two biogeographic zones and ranging from the coast to high mountains, the Bach Ma National Park is considered to be a biodiversity hotspot (see Kunich 2003; Matusch 2014; Adler et al. 2016; Ngoc et al. 2016).

Malaise trap samples usually contain a wide array of insect taxa, but are dominated by Diptera and Hymenoptera (see e.g. Gressitt and Gressitt 1962; Matthews and Matthews 1971; Moeed and Meads 1987; Karlsson et al. 2005; Skvarla 2015; Geiger et al. 2016). However, a number of Lepidoptera species were caught as bycatch. On the one hand, extensive molecular research on butterflies and moths from Malaise trap samples preserved in ethanol has proven successful (see e.g. Tänzler et al. 2012; Aagaard et al. 2016; Geiger et al. 2016; Morinière et al. 2016; Cancian de Araujo et al. 2017; Cancian de Araujo et al. 2018), but on the other hand, morphological studies have been hampered, mainly because Lepidoptera are regarded as being unsuitable for dry-mounting after fixation in ethanol (see e.g. Walker and Crosby 1988; Brown 2005; Krogmann and Holstein 2010). Nevertheless, as reported earlier (see e.g. Owen 1971; Walker 1978; Covell and Freytag 1979; Sivinski 2014), Malaise traps have been successfully employed in sampling and in the general evaluation of butterflies based on external morphology without setting the specimens. In the present study, we aimed at developing methods to prepare Lepidoptera preserved in ethanol for routine external morphological analysis, whereby the family Geometridae has been selected as a target group.

### Use of Lepidoptera preserved in ethanol for morphological studies

**Figure 1.**
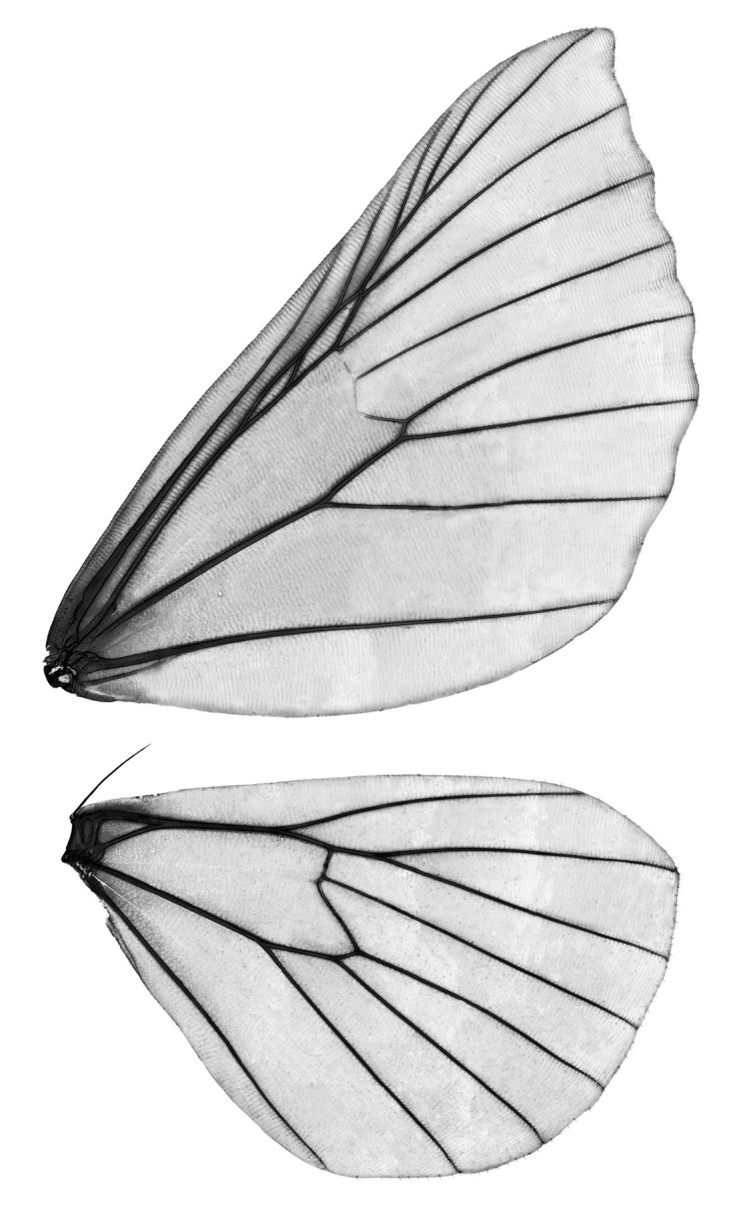
Larentiinae (Lepidoptera, Geometridae): male, wing venation (specimen originally preserved in 80% ethanol).

**Figure 2.**
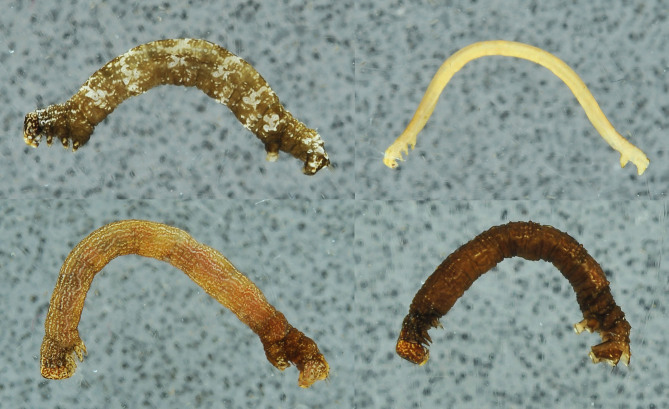
'VIETBIO' Malaise trap sample: Lepidoptera larvae preserved in 80% ethanol.

- **Genitalia slides. **Malaise traps were reported to deliver excellent material for slide mounting of Macrolepidoptera and species identification based on the genitalia characters (Schmidt 2016). The method of enzymatic digestion of the abdomen (see Knölke et al. 2005) for obtaining DNA for sequencing and maceration of tissues for the preparation of genitalia is also applicable for specimens that were preserved in ethanol. Furthermore, material from Malaise trap projects stored in ethanol have been used to examine the genitalia of Microlepidoptera, including Nepticulidae (see e.g. van Nieukerken et al. 2016).
- **Wing venation. **Preparation of wing venation slides of Lepidoptera usually involves separation of the wings from the body, transferring them to ethanol, bleaching with sodium hypochlorite solution, and carefully removing the scales with a thin brush. If a specimen is preserved in ethanol, bleaching is not necessary as scales are detached easily. It only takes a little effort to remove the scales without damaging the wings (see Fig. 1).
- **Study of the larvae.** A standard procedure to preserve larvae for further study requires fixation with boiling water or preservative fluid (see e.g. Peterson 1962; Stehr 1987). After that, the larvae can be stored in 70-80% ethanol. However, larvae that were collected with Malaise traps and subsequently preserved in ethanol are perfectly suitable for eventual morphological studies (Fig. 2).
- **Study of the eggs.** Eggs for morphological studies can be obtained from dry museum specimens by enzymatic digestion of the abdomen (see Junker et al. 2006). The same method can be used for the Malaise trap samples preserved in ethanol after drying the specimens (Schmidt unpublished data). Wet specimens were not tested.
- **Study of the genitalia musculature.** Characters of the skeleton and the musculature of male genitalia have proven to be useful for the higher classification of butterflies and moths and samples of ethanol-preserved Lepidoptera have been used for this type of study (see e.g. Schmidt 2013). Malaise traps were occasionally employed for sampling specimens for morpho-functional studies of male genitalia (Schmidt 2014a, Schmidt 2014b, Schmidt 2017). Thoracic skeletomuscular characters are not considered in the present report although thorax and legs were found to be well preserved in Malaise trap samples.

## Material and methods

The sampling regime consisted of four Malaise traps that were operated for 6-9 days across a range of different habitats in the Bach Ma National Park (16.19°N, 107.85°E) in central Vietnam at altitudes between 520 and 1400 m a.s.l. in June 2018. The collecting efforts resulted in ten samples with specimens preserved in 80% ethanol. Geometrid moths were carefully removed from the samples using featherweight forceps. Additionally, several butterfly specimens of the diverse family Nymphalidae and representatives of the families Erebidae, Noctuidae and Pyralidae were selected for pinning and setting. Altogether, about 40 specimens from Vietnam were tested. To assess the suitability of specimens from older Malaise trap samples, about 40 Lepidoptera specimens including Nymphalidae, Hesperiidae, Geometridae, Erebidae, Noctuidae, Pyralidae and Tortricidae from Malaise trap samples collected in the Bavarian Forest National Park (48.96°N, 13.39°E) in Germany in the years 2005 and 2011 were selected for setting. The samples were preserved in 300-500 ml plastic jars with plenty of 80% ethanol and stored in a refrigerator at temperatures between 4°C and 7°C. The wing expanse (twice the distance from midthorax to the forewing apex) of the selected specimens was 15-90 mm. Smaller moths were not tested. The material is deposited in Munich (SNSB – Zoologische Staatssammlung München, Germany) and in Hanoi (Vietnam National Museum of Nature, VAST, Hanoi, Vietnam).

A list of tools required for setting Lepidoptera is provided by Schmidt et al. (2017). A range of preparation methods have been described before (e.g. Klots 1973; Common and Waterhouse 1972; Gibb and Oseto 2006; Resh and Carde 2009; Eymann et al. 2010; Häuser and Riede 2015) or they are available as online resources (e.g. Wheeler et al. 2001; Warren 2015).

## The workflow protocol

**Figure 3.**
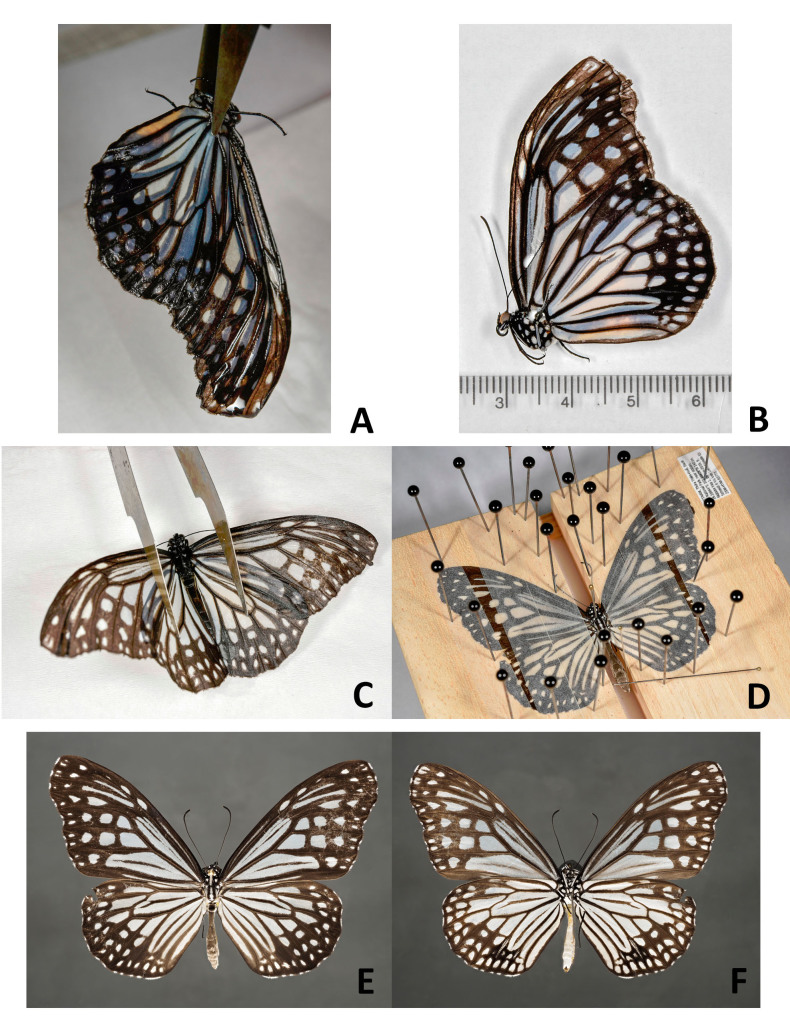
Setting a butterfly (Lepidoptera, Nymphalidae); **A**. taking the specimen out of the ethanol; **B.** drying the specimen on a piece of filter paper; **C**. moving left and right pairs of wings apart; **D.** setting the specimen using a spreading board; **E.** dried specimen, wings above; **F.** dried specimen, wings underneath.

**Figure 4.**
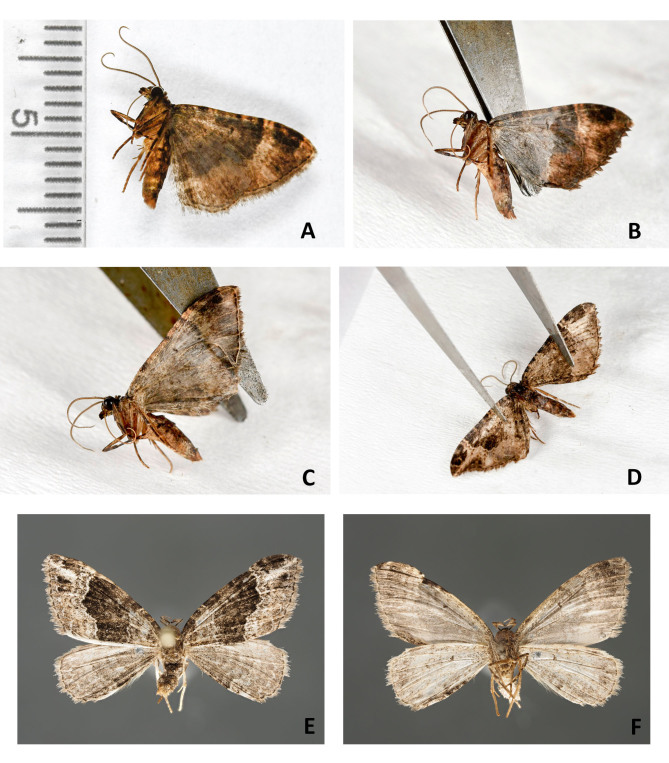
Setting a larentiine moth (Lepidoptera, Geometridae); **A.** drying the specimen on a piece of filter paper; **B-D.** moving left and right pairs of wings apart; **E**. dried specimen, wings above; **F.** dried specimen, wings underneath.

**Figure 5.**
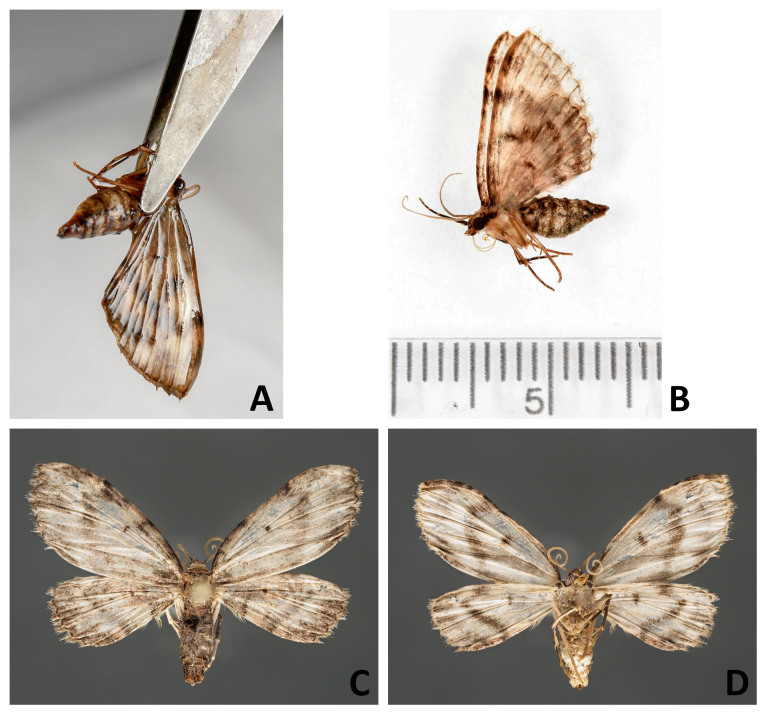
Setting a larentiine moth (Lepidoptera, Geometridae); **A**. taking the specimen out of the ethanol; **B.** drying the specimen on a piece of filter paper; **C.** dried specimen, wings above; **D.** dried specimen, wings underneath.

1. From a sample preserved in ethanol, preferably select a specimen that has its wings folded together over its back. If the specimen has its wings spread or in a downward position, bring the wings into position so that they are dorsally folded with the help of featherweight forceps before removing the specimen from the ethanol. This procedure should be easy with recently sampled specimens. If the specimens have been preserved in ethanol for a long time, they are less flexible. In this case, leave the wings in the downward position.
2. Using forceps, take the specimen out of the ethanol holding it by the thorax with the wings directed down (Fig. 3A; Fig. 5A). Let the ethanol run from the wing apices. Gently lay the specimen on filter paper and allow to dry for 2-7 minutes depending on the size of the specimen and the thickness of the wings (Fig. 3B; Fig. 4A; Fig. 5B). Do not let the specimen dry completely.
3. With a pair of forceps, carefully move left and right pairs of wings apart (Fig. 3C; Fig. 4B-D). Using two pairs of forceps, move the hind- and forewings apart starting from the wing base.
4. Put the specimen on a piece of plastazote foam and, holding the wings with a pair of forceps (see Fig. 3C; Fig. 4D), put an insect pin through the middle of the thorax. In case the specimen had the wings folded over its back, force the wings down with forceps after pinning.
5. Spread the wings using a spreading board following the standard procedure (Fig. 3D).
6. Leave the specimen on a spreading board for at least one week. Insects that were originally preserved in ethanol take less time to dry than the fresh ones.

## Results and discussion

**1.** The present study allowed us to obtain valuable insights into the challenges but also the opportunities of using Lepidoptera from Malaise trap samples for morphological study. The rapid global biodiversity loss (see Cardinale et al. 2012; Ceballos et al. 2015), combined with the lack of research funding for fieldwork in regions with a poorly known fauna (Barber et al. 2014; Titley et al. 2017), underpins the urgent need for new concepts and protocols that allow the efficient and comprehensive analysis of Malaise trap and other samples obtained during collecting trips that are often undertaken under financial constraints and in a short time frame. Our study presents a protocol that uses butterflies and moths from Malaise traps that have usually been neglected as a source for taxonomic studies.

**Figure 6.**
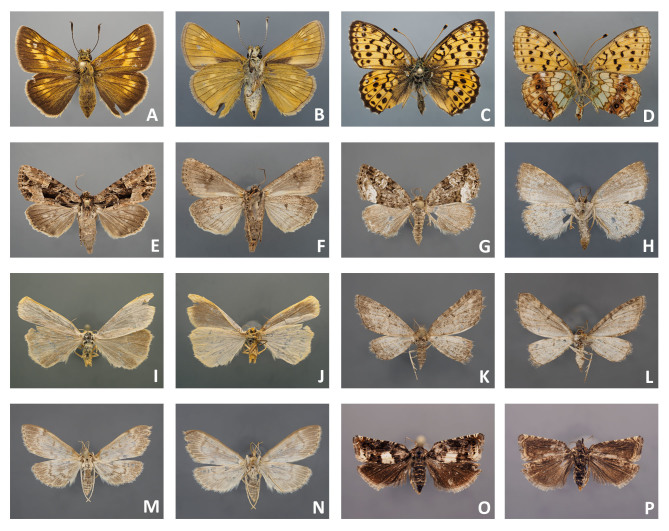
Mounted Lepidoptera from Malaise trap samples (Bavaria, 2005); **A-D. **butterflies; **E-L.** larger moths; **M-P.** smaller moths.

**Figure 7.**
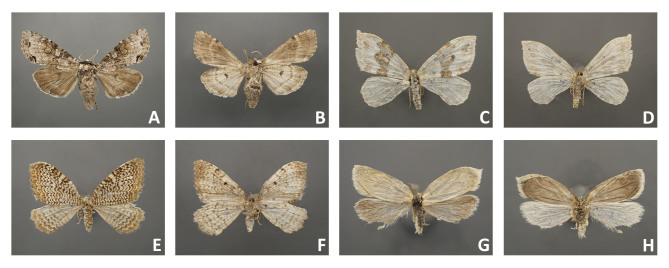
Mounted Lepidoptera from Malaise trap samples (Bavaria, 2011); **A-F.** larger moths; **G-H.** smaller moths.

**Figure 8.**
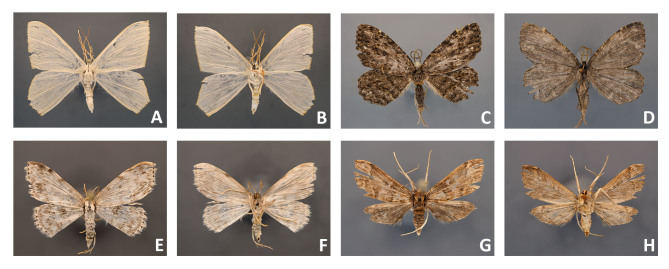
Mounted Lepidoptera from Malaise trap samples (Vietnam, 2018); **A-F.** larger moths; **G-H.** smaller moths.

**2.** Best results were achieved when mounting specimens with robust wings and contrasting wing patterns, regardless of the family (Fig. 3E-F; Fig. 6A-H; Fig. 7A-B; Fig. 8C-D). All ten specimens of nymphalid and hesperiid butterflies and all seven specimens of noctuid moths tested in the present study, even after several years of storage, looked like freshly collected specimens after the setting procedure. In terms of standard morphological descriptions based on external morphology, there was hardly any difference between freshly collected specimens and specimens that were preserved in ethanol.

**3.** Not all specimens were in perfect condition after sampling in ethanol and mounting. Light coloured wings of small moths fade and lose their scales easily, even if taken from recent Malaise trap samples (Fig. 5C-D; Fig. 8E-H). Specimens with thin broad wings are not quite suitable for setting and their wings are often cambered after removal from spreading boards (Fig. 6I-J). It is known that green specimens do not retain the original colour and turn whitish when preserved in ethanol (Fig. 8A-B). Nonetheless, more than half of the pyralid and tortricid specimens were good enough for study of morphology after mounting.

**4.** Comparison of specimens from the older (2005 and 2011) and recent (2018) Malaise trap samples (see Fig. 6A-P; Fig. 7A-H; Fig. 8A-H) showed that specimens from recent samples were easier to handle and some specimens were less worn than the others, but even older specimens that were collected in 2005 were mounted successfully.

**5.** Specimens of different subfamilies of Geometridae, including Geometrinae, Ennominae, Larentiinae, and Sterrhinae were tested. All ten specimens of boarmiine ennomine moths (e.g. Fig. 8C-D) included in the present study and all five thick-bodied geometrine specimens were good enough to be described based on morphology. However, many species of Larentiinae and Sterrhinae have thinner wings. They lose their scales easily (see Fig. 7C-D; Fig. 8E-F) and are difficult to mount if preserved in ethanol. Only specimens with contrasting wing patterns were suitable for standard morphological descriptions (e.g. Fig. 4E-F; Fig. 7E-F). Testing of small Microlepidoptera was beyond the frame of the present report.

**6.** In deciding whether it is worth processing the specimens in ethanol for faunistic studies, a closer look has been taken at the target group, larentiine geometrid moths. Parallel to targeted light-trapping which has been carried out for 12 nights, standard Malaise trap sampling was performed for 6 to 9 days. About 60 species of larentiine moths have been collected using light sources, whereas 10 species were sorted from the Malaise trap samples, of which at least one species was not taken at a light trap.
